# Single-cell RNA-seq reveals the role of YAP1 in prefrontal cortex microglia in depression

**DOI:** 10.1186/s12883-024-03685-1

**Published:** 2024-06-07

**Authors:** Fenghui Ma, Hongjun Bian, Wenyan Jiao, Ni Zhang

**Affiliations:** 1 Department of Health Management, Tangdu Hospital, Fourth Military Medical University, Xi’an, Shaanxi 710038 China; 2 Department of Pediatrics, the Second Affiliated Hospital of Xi’an Jiaotong University, Xi’an, Shaanxi 710000 China; 3 Department of Psychiatry, Shaanxi Provincial People’s Hospital, Xi’an, Shaanxi 710068 China; 4 Department of Mental Health, Xi’an ZhongShengKaiXin Technology Development Co., Ltd., Xi’an, Shaanxi 710000 China

**Keywords:** Depression, Prefrontal cortex, Microglia, Single-cell RNA-seq, YAP1

## Abstract

**Background:**

Depression is a complex mood disorder whose pathogenesis involves multiple cell types and molecular pathways. The prefrontal cortex, as a key brain region for emotional regulation, plays a crucial role in depression. Microglia, as immune cells of the central nervous system, have been closely linked to the development and progression of depression through their dysfunctional states. This study aims to utilize single-cell RNA-seq technology to reveal the pathogenic mechanism of YAP1 in prefrontal cortex microglia in depression.

**Methods:**

Firstly, we performed cell type identification and differential analysis on normal and depressed prefrontal cortex tissues by mining single-cell RNA-seq datasets from public databases. Focusing on microglia, we conducted sub-clustering, differential gene KEGG enrichment analysis, intercellular interaction analysis, and pseudotime analysis. Additionally, a cross-species analysis was performed to explore the similarities and differences between human and rhesus monkey prefrontal cortex microglia. To validate our findings, we combined bulk RNA-Seq and WGCNA analysis to reveal key genes associated with depression and verified the relationship between YAP1 and depression using clinical samples.

**Results:**

Our study found significant changes in the proportion and transcriptional profiles of microglia in depressed prefrontal cortex tissues. Further analysis revealed multiple subpopulations of microglia and their associated differential genes and signaling pathways related to depression. YAP1 was identified as a key molecule contributing to the development of depression and was significantly elevated in depression patients. Moreover, the expression level of YAP1 was positively correlated with HAMD scores, suggesting its potential as a biomarker for predicting the onset of depression.

**Conclusion:**

This study utilized single-cell RNA-seq technology to reveal the pathogenic mechanism of YAP1 in prefrontal cortex microglia in depression, providing a new perspective for a deeper understanding of the pathophysiology of depression and identifying potential targets for developing novel treatment strategies.

**Supplementary Information:**

The online version contains supplementary material available at 10.1186/s12883-024-03685-1.

## Introduction

Depression is a common and severe mental disorder characterized by persistent low mood, loss of interest, and cognitive decline [1]. The prefrontal cortex, as a crucial region of the brain involved in emotional regulation, cognitive function, and behavioral control, plays a pivotal role in the pathogenesis of depression [2]. Recent advancements in single-cell RNA-seq technology have enabled unprecedented precision in exploring transcriptional changes in different cell types within the prefrontal cortex, thereby shedding light on the cellular and molecular mechanisms underlying depression [3–5].

Microglia, as resident immune cells of the central nervous system, not only maintain brain homeostasis but also participate in the pathological processes of various neuropsychiatric diseases [6]. Mounting evidence suggests a close association between microglial activation and dysfunctional states and the onset of depression [7, 8]. However, the specific mechanistic role of microglia in depression remains incompletely understood.

YAP1 (Yes-Associated Protein 1) is an essential transcriptional co-activator that plays a critical role in the Hippo signaling pathway, regulating multiple biological processes such as cell proliferation, differentiation, and apoptosis [9, 10]. Recent studies have implicated YAP1 in the development and pathogenesis of the nervous system [11]. Nonetheless, the expression changes of YAP1 in depression and its relationship with microglial function remain unexplored.

This study aims to utilize single-cell sequencing technology to systematically explore the transcriptional changes of microglia in the prefrontal cortex in depression and delve into the pivotal role of YAP1 therein. The single-cell RNA-seq data analyzed in this research was originally published in *Nature Neuroscience* in 2020 [12], where the authors discovered the significant role of oligodendrocyte precursor cells and deep layer excitatory neurons in depression. However, microglia, as an essential cell type in the nervous system, were not fully investigated in that study. Therefore, we re-analyzed these single-cell RNA-seq data to understand which genes in microglia have changed during the development of depression. Building upon this foundation, we employed various bioinformatics methods, including bulk RNA-seq and Weighted Gene Co-Expression Network Analysis (WGCNA), to identify YAP1 as a key gene closely associated with the pathogenesis of depression. Finally, the correlation between YAP1 expression levels and the severity of depression was validated, providing a new perspective and approach for a deeper understanding of the pathogenesis of depression.

## Methods

### Single-cell RNA-seq data processing

The single-cell RNA-seq dataset GSE144136 was obtained from public databases, containing single-cell transcriptome data from prefrontal lobe tissues of 17 healthy individuals and 17 patients with depression [12]. Data preprocessing was performed using the Seurat R package, including quality control, normalization, dimensionality reduction (using PCA), and clustering (employing a graph-based clustering method). Following clustering, the data was visualized using t-SNE, and individual cell populations were annotated using the SingleR package and CellMarker tool to determine their cell types.

### Pseudotime analysis

To explore the trajectory of cell development, pseudotime analysis was conducted. The Monocle R package was used to sort the single-cell data, simulating the process of cell development. By analyzing trends in gene expression changes, different developmental states and corresponding signaling pathways were identified.

### Ligand-receptor analysis

To investigate intercellular communication, ligand-receptor analysis was performed. The Cellchat R package was utilized to analyze ligand-receptor interactions among various cell types. This package predicts intercellular communication networks based on single-cell RNA-seq data and identifies crucial ligands and receptors. Differences in ligand-receptor interactions between depressed and normal prefrontal cortex tissues were compared, exploring their potential biological significance.

### Cross-species single-cell analysis

To identify key genes contributing to depression, a cross-species analysis comparing human and rhesus monkey prefrontal cortex tissues was conducted. Single-cell RNA-seq data from both species were obtained from public databases and integrated and normalized using the Seurat R package. The correlation between cell types across species was examined using scMerge packages. Additionally, a differential analysis was performed on microglia from the two species to reveal similarities and differences.

### Bulk RNA-Seq and WGCNA analysis

To investigate molecular marker changes in the prefrontal cortex of depression patients, bulk transcriptome datasets (GSE98793, GSE76826) were downloaded from the GEO database [13, 14]. Differential expression analysis was performed using the Deseq2 function. Additionally, WGCNA was conducted on another GEO microarray dataset to identify gene modules and key genes associated with depression pathogenesis.

### Validation and analysis of YAP1

Based on the results of bulk RNA-Seq, WGCNA, and single-cell RNA-Seq, differentially expressed genes were screened, and YAP1 was identified as a key molecule through PPI network analysis. To validate YAP1’s expression in depression, additional datasets (GSE32280) were utilized, and YAP1 expression patterns were observed in single-cell and microglial data [15]. Furthermore, the Allen ISH brain atlas(https://mouse.brain-map.org/) was consulted to confirm YAP1 expression in the prefrontal cortex. Allen ISH brain atlas is a publicly available in situ hybridization atlas that allows researchers to access the expression of mRNA in mouse brain tissue [16].

### Sample collection and validation

To further examine the relationship between YAP1 and depression, 5mL of fasting venous blood samples were collected from depression patients. The serum YAP1 levels were quantified using the ELISA, and HAMD [17] scores were used to assess the severity of depression. The relationship between YAP1 expression levels and depression severity was assessed through statistical analyses, including correlation analysis and ROC curve analysis. Determine whether YAP1 is an independent risk factor for depression through univariate and multivariate analysis.

## Results

### Single-cell clustering reveals cell types in the prefrontal cortex

**Fig. 1:**
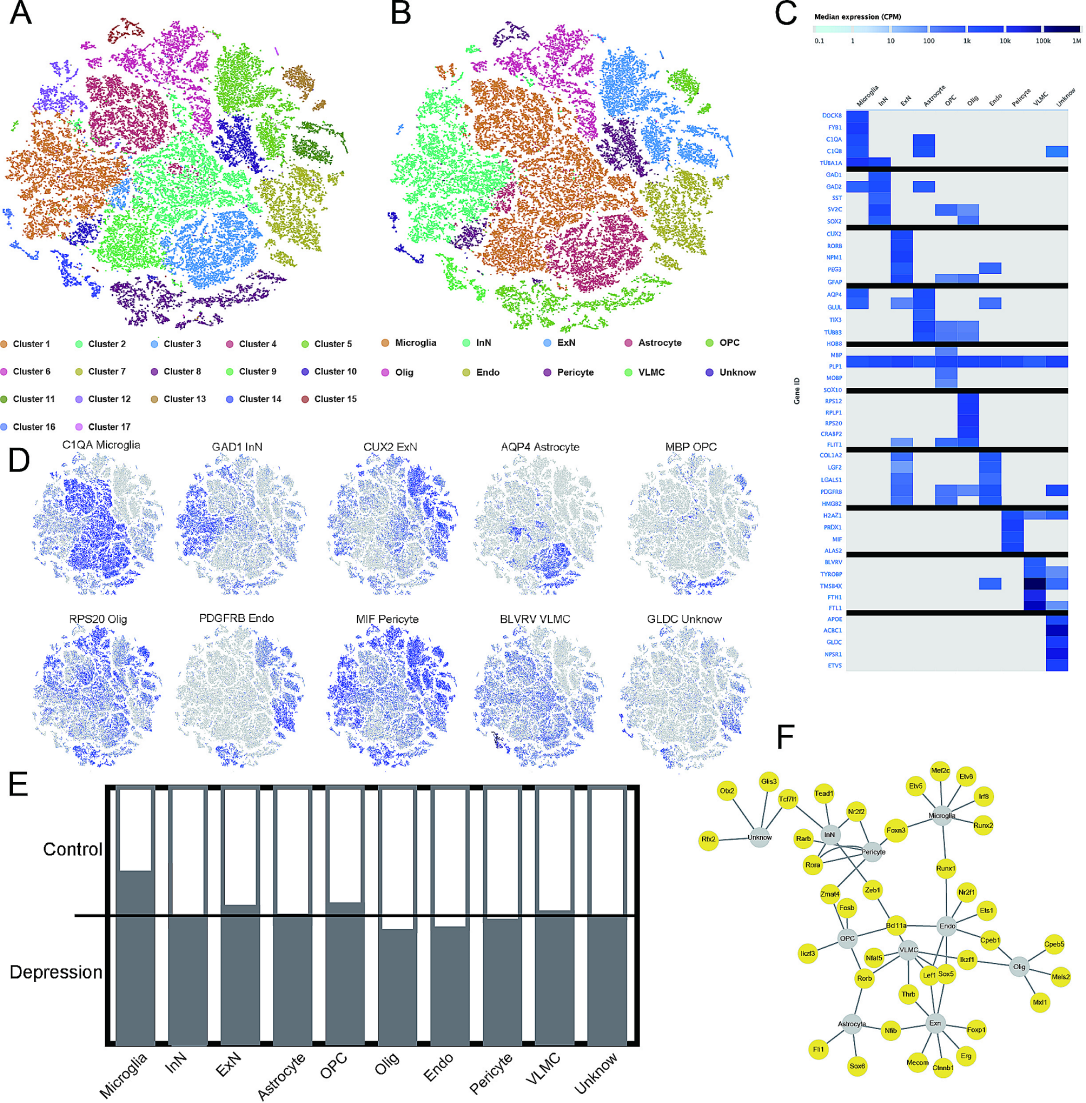
Single-cell transcriptome data from prefrontal cortex tissue and depressive prefrontal cortex tissue. (**A**) t-SNE plot showing cell clustering in normal and depressive prefrontal cortex tissues, resulting in 17 clusters; (**B**) Identification of 10 cell types; (**C**) Heatmap of the top 5 marker genes for each cell type; (**D**) t-SNE plot displaying typical marker genes for each cell type; (**E**) Changes in the proportion of each cell type in control and depressive prefrontal cortex tissues; (**F**) PPI analysis of transcription factors among marker genes for each cell type

By mining the single-cell RNA-seq dataset GSE144136 from public databases, which includes normal prefrontal cortex tissue and depressive prefrontal cortex tissue, we obtained a total of 17 clusters through dimensionality reduction and clustering processes using the Seurat package, as shown in the t-SNE plot (Fig. 1A). Each cluster represents a group of cells with similar gene expression patterns, which are clustered together due to their shared marker genes. Different clusters may belong to the same cell type, such as neurons. Neurons can be subdivided into many subtypes based on neurotransmitters, which means neurons with the same neurotransmitter may be assigned to the same cluster. However, when annotating these cells, we collectively refer to neurons with different neurotransmitters as excitatory neurons or inhibitory neurons. The same applies to microglia and astrocytes. Therefore, using like SingleR packages and CellMarker websites, we annotated the cell types of these clusters and merged some clusters of the same cell type in Fig. 1A. As a result, we obtained a total of 10 cell types (Fig. 1B): Microglia, Inhibitory Neuron (InN), Excitatory Neuron (EnN), Astrocyte, Oligodendrocyte Precursor Cell (OPC), Oligodendrocyte (Olig), Endothelial Cell (Endo), Pericyte, Vascular Leptomeningeal Cell (VLMC), and Unknown (Fig. 1B). Compared to the published results, the current study identified a greater number of cell types, reaching a total of 10, while the published study reported 7 types [12]. The additional three cell types in our study are “VLMC,” “Pericyte,” and “Unknown”. We speculate that this difference is attributed to the inconsistent cell filtering conditions used in the analyses. We then displayed the top 5 marker genes for each cell type using a heatmap (Fig. 1C) and showed the typical marker genes for each cell type using a t-SNE plot, and these ten t-SNE plots represent ten marker genes, which correspond to ten different cell types, respectively (Fig. 1D). A bar chart was used to illustrate the proportion of each cell subtype in the Control group (healthy prefrontal cortex) and the Depression group (depressive prefrontal cortex), the results showed that the proportion of Microglia cell type changed most significantly, decreasing from 69.87% in the depression group to 30.13% in the control group, while no significant changes were observed in other cell types (Fig. 1E). PPI network analysis was conducted to examine the transcription factors among the marker genes of each cell type (Fig. 1F), indicating close relationships between Microglia and multiple transcription factors such as Runx2, Etv5, and Etv6, while other cell types were also closely associated with different transcription factors, suggesting significant transcriptional differences across cell types.

### Single-cell subclustering of microglia

**Fig. 2:**
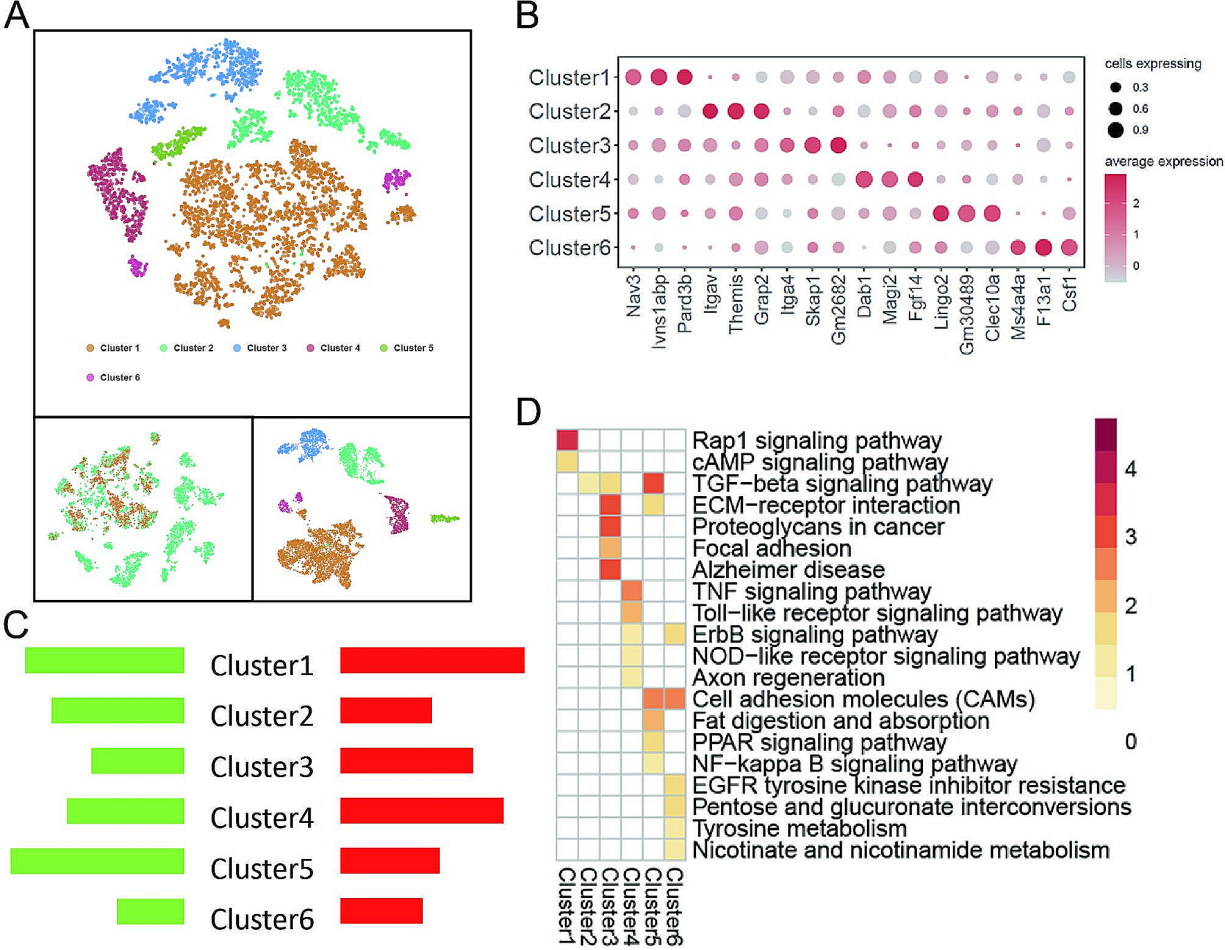
Single-cell transcriptome data focusing on Microglia from normal and depressive prefrontal cortex tissues. (**A**) t-SNE and UMAP plots showing the results of Microglia subclustering; (**B**) Dotplot displaying the top 3 marker genes for each cluster; (**C**) Statistical graph showing the number of differential genes for each cluster; (**D**) Heatmap of KEGG enrichment analysis for differential genes across all clusters

Based on the results from the previous section, which showed significant changes in Microglia cells in depressive tissues, we further subclustered these cells, identifying six clusters. These cell types were derived from both the Control group (normal prefrontal cortex) and the Depression group (depressive prefrontal cortex), exhibiting high homogeneity between groups (Fig. 2A). A Dotplot was used to display the top 3 marker genes for each cluster (Fig. 2B). Differential analysis was performed to identify differentially expressed genes between normal and depressive prefrontal cortex tissues for each cluster, and Fig. 2C shows the number of differentially expressed genes among different microglia subpopulations, with cluster 1 showing the highest number of both upregulated and downregulated genes. Different numbers of differentially expressed genes suggest that these clusters may play distinct roles in depression. KEGG enrichment analysis of these differential genes revealed extensive changes in signaling pathways, including Rap1 signaling pathway, CAMP signaling pathway, TGF-beta signaling pathway, ECM-receptor interaction, and others (Fig. 2D).

### Ligand-Receptor interaction analysis and pseudotime analysis

**Fig. 3:**
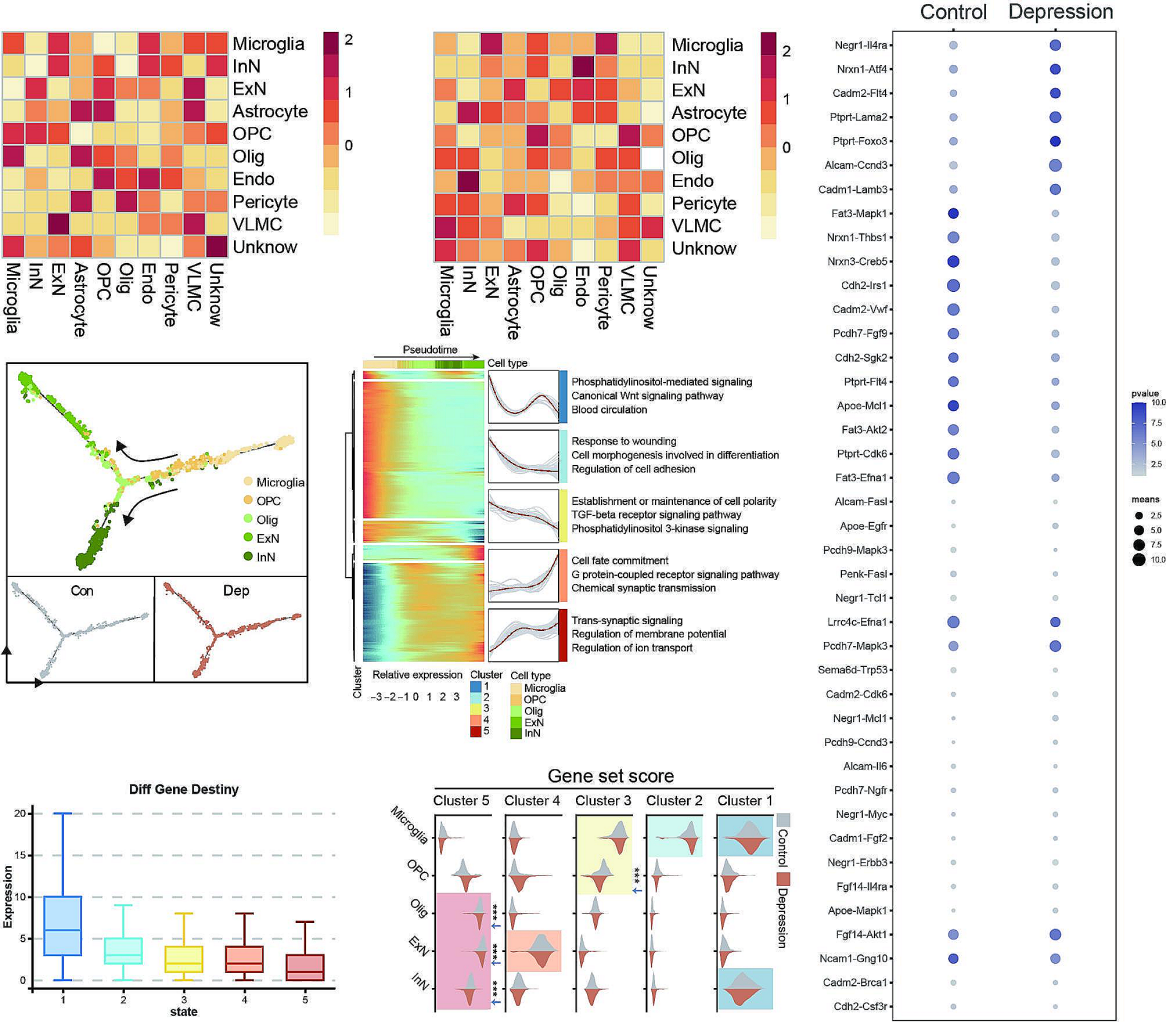
Ligand-receptor interaction analysis and pseudotime analysis. (**A**) Ligand-receptor interaction analysis in depressive prefrontal cortex tissue; (**B**) Ligand-receptor interaction analysis in normal prefrontal cortex tissue; (**C**) Dotplot showing altered ligand-receptor pairs between Microglia and excitatory/inhibitory neurons; (**D**) Pseudotime analysis of cell development, illustrating Control and Depression groups; (**E**) Enrichment analysis of genes in different clusters; (**F**) Diff Gene Destiny scoring results for different Stats; (**G**) Comparison of Gene set scores between Control and Depression groups across clusters

We employed the Cellchat package to analyze the ligand-receptor interactions among cells (Microglia, InN, ExN, Astrocyte, OPC, Olig, Endo, Pericyte, VLMC, Unknown) in depressive and normal prefrontal cortex tissues. The results indicated notable changes in the number of ligand-receptor pairs between microglia and inhibitory neurons. Specifically, in the Control group, expressions of Fat3-Mapk1, Nrxn1-Thbs1, Nrxn3-Creb5, and others were significantly elevated, while in the Depression group, expressions of Negr1-1l4ra, Nrxn1-Atf4, Cadm2-Fit4, and others were notably reduced (Fig. 3C). Subsequently, we performed pseudotime analysis on these cells, simulating five distinct developmental states corresponding to different gene expression trends and signaling pathways (Fig. 3D and E). The Diff Gene Destiny scores for different developmental states suggested a higher level of variation in Stat1 compared to other Stats (Fig. 3F), and the Gene set score results indicated varying degrees of changes in Olig, ExN, and InN cell subpopulations between depressive and normal prefrontal cortex tissues (Fig. 3G).

### Cross-species analysis reveals similarities and differences between humans and rhesus monkeys

**Fig. 4:**
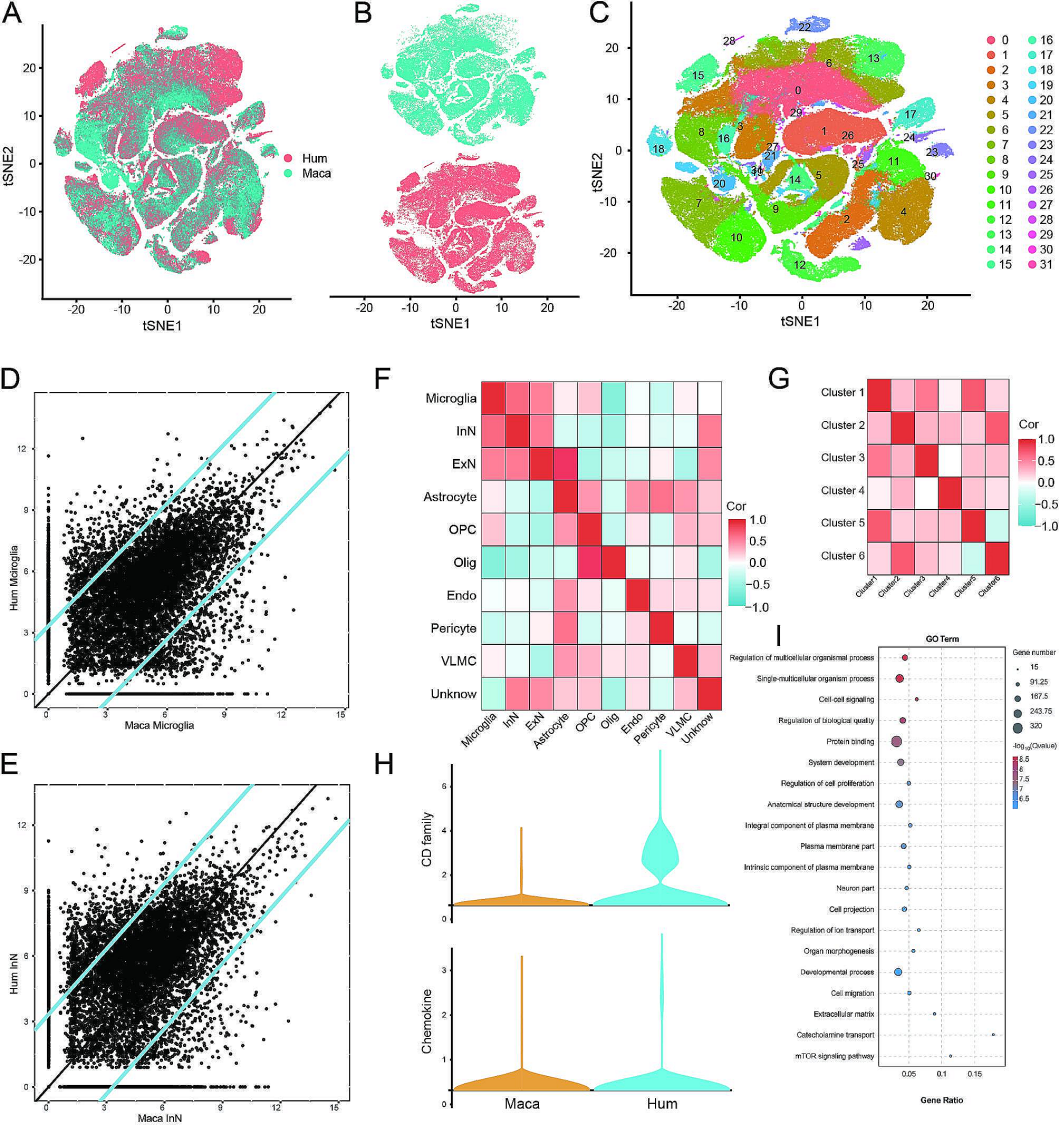
Cross-species analysis comparing humans and rhesus monkeys. (**A**) t-SNE plot showing the combined analysis of normal prefrontal cortex tissues from both species; (**B**) Separate t-SNE plots for each species; (**C**) Identification of a total of 32 clusters; (**D-E**) Correlation comparison of Microglia and inhibitory neurons between the two species; (**F**) Heatmap showing the correlation comparison among all 10 cell types; (**G**) Heatmap comparing the correlation among 6 Microglia subpopulations; (**H**) Comparison of CD family and Chemokine expressions between the two species; (**I**) GO enrichment analysis of differential genes between the two species

The above results demonstrate the significant importance of microglia in the prefrontal cortex tissue of humans with depression. However, preclinical studies are generally conducted based on primates, and such species differences are the main reason why many treatments are effective in animal experiments but not in clinical use. Therefore, clarifying the differences and conservativeness between species has certain value for developing effective depression treatments. Therefore, we conducted a cross-species analysis comparing normal prefrontal cortex tissues from humans and rhesus monkeys (GSE201687) (Fig. 4A, B). This analysis resulted in a total of 32 clusters (Fig. 4C) and the identification of 10 cell types, similar to previous results. Focusing on the critical roles of Microglia in depression, we compared the correlation of these two cell types between humans and rhesus monkeys using the AUC package (Fig. 4D). In addition, based on the interaction analysis in Fig. 3A-B, a significant cellular interaction between microglia and inhibitory neurons was found. Therefore, this study confirms that inhibitory neurons also exhibit a high degree of conservation across species (Fig. 4E). The results showed a significant correlation between the two species for both cell types. We further extended this comparison to all identified cell types (Fig. 4F), revealing strong correlations within the same cell types but relatively weaker correlations between different types. Despite subclustering, the Microglia subpopulations exhibited strong correlations among themselves, indicating evolutionary conservation (Fig. 4G). Additionally, we performed a differential analysis comparing Microglia cells between the two species, finding that the CD family was significantly higher in human prefrontal cortex tissue, while there was no statistical difference in Chemokine expression (Fig. 4H). Furthermore, differences exist between the two species as well. GO analysis of the differential genes between the two species revealed changes in signaling pathways such as Regulation of multicellular organismal process, Single-multicellular organism process, and Cell-cell signaling (Fig. 4I). This suggests that there are also some evolutionary differences between the two species.

### Bulk RNA-Seq and WGCNA reveal key genes in depression

**Fig. 5:**
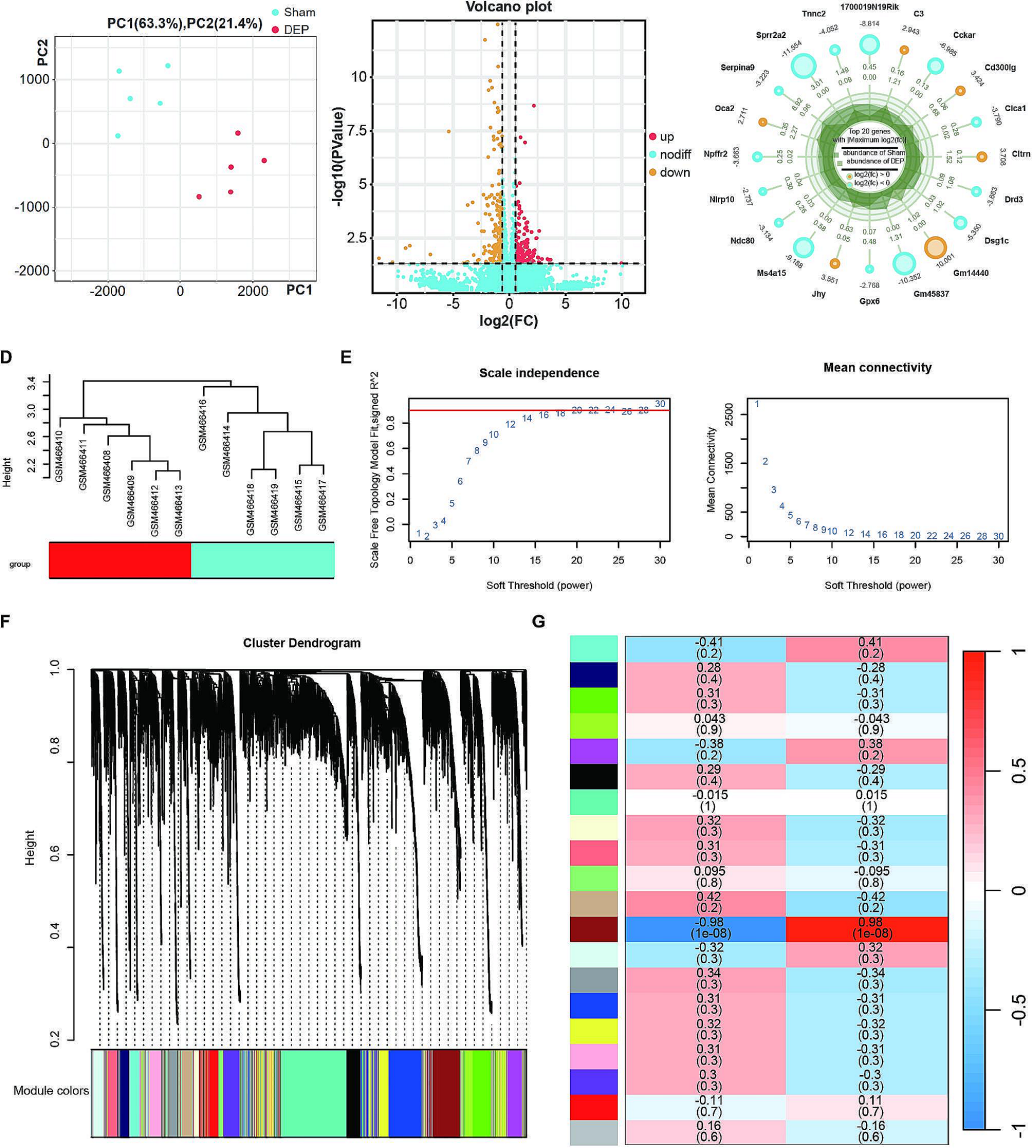
Bulk RNA-seq and WGCNA reveal key genes in depression. (**A**) PCA analysis comparing control and depression groups; (**B**) Volcano plot showing differentially expressed genes, with red dots representing upregulated genes, yellow dots representing downregulated genes, and blue dots representing non-differential genes; (**C**) Display of the top 10 differentially expressed genes; (**D**) Clustering of expression profiles in two sample groups; (**E**) Analysis of scale-free fit index and mean connectivity for different soft thresholds in WGCNA enrichment analysis; (**F**) Division of differentially expressed genes into distinct gene modules and their clustering results in WGCNA enrichment analysis; (**G**) Correlation between each gene module and depression in WGCNA enrichment analysis, with red indicating a positive correlation and blue indicating a negative correlation

To investigate molecular marker changes in the prefrontal cortex of depression patients, we analyzed bulk transcriptome datasets (GSE98793, GSE76826) obtained from the GEO database, comparing normal volunteers with depression patients. PCA analysis was performed to assess clustering (Fig. 5A), followed by bioinformatic analysis using the Deseq2 function. We identified 145 differentially expressed genes (|log2FC|>1.2 and *P* < 0.05), including 86 downregulated and 59 upregulated genes (Fig. 5B). The results of enrichment analysis of these differential genes showed significant changes in inflammation-related signaling pathways such as Epstein-Barr virus infection, Hematopoietic cell lineage, and NF-kappa B signaling pathway (Figure S1). These results are similar to those obtained from the differential analysis of microglia in scRNA-seq (Fig. 2D), emphasizing the important role of inflammatory response in depression. The top 20 downregulated and upregulated genes are shown in Fig. 5C. Additionally, we conducted WGCNA enrichment analysis on the GEO chip dataset from patients with conventional depression. Using R software, we calculated a soft threshold of β = 21 (Fig. 5D-E) and constructed a co-expression matrix network based on this threshold. The main purpose of soft threshold screening is to make the gene regulatory network more consistent with the distribution characteristics of a scale-free network. Specifically, the soft threshold is obtained by performing a power operation on the correlation of gene expression, and the value of this power is the soft threshold. By adjusting the size of the soft threshold, we can change the weight distribution of edges in the network, making the network structure more consistent with the characteristics of a scale-free network. In this way, we can construct a robust gene regulatory network that not only reflects real biological processes but also possesses robustness. Figure 5F is a gene clustering dendrogram, the purpose of which is to group genes with similar expression patterns in the prefrontal cortex tissue through clustering algorithms, forming gene modules, thereby revealing potential functional associations among genes. Figure 5G is a heatmap of inter-module correlations, the purpose of which is to demonstrate the strength of correlation between different gene modules and depressive symptoms. This analysis aids in understanding the interactions and regulatory relationships among modules, providing guidance for further functional research and applications. The MEbrown module is chosen because the genes under this module have the closest relationship with depression, with a correlation coefficient of 0.98, which is much higher than other modules. Therefore, through WGCNA analysis, we found that 168 genes in MEbrown are closely related to depression.

### YAP1 identified as a key molecule in the development of depression

**Fig. 6:**
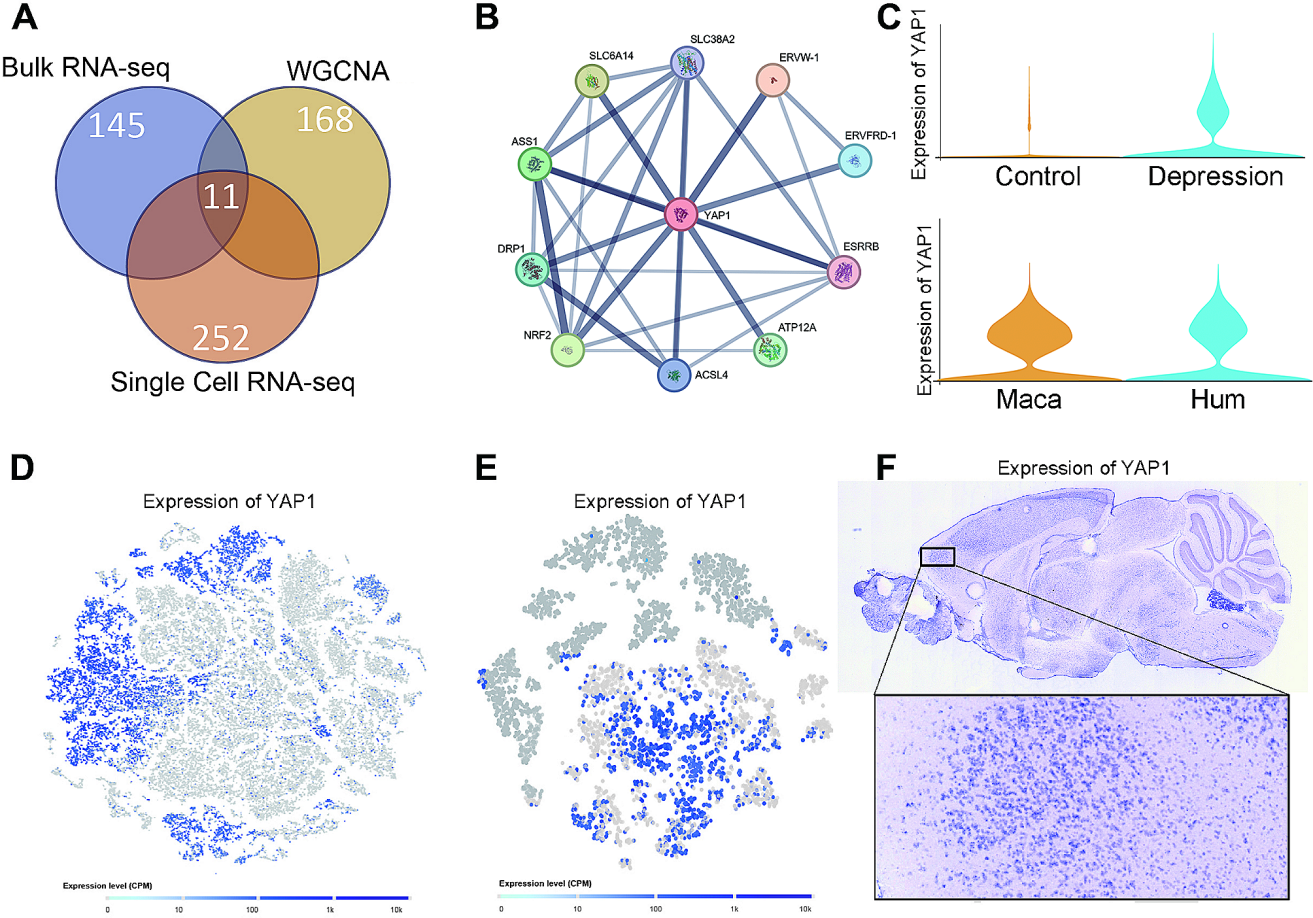
YAP1 Identified as a Key Molecule in the Development of Depression. (**A**) Venn diagram analysis of differentially expressed genes identified through Bulk RNA-Seq of normal and depressed prefrontal cortex tissues, WGCNA analysis, and Single-cell RNA-Seq. (**B**) PPI network analysis targeting the 11 genes. (**C**) Validation of YAP1 expression levels in the GEO microarray dataset and single cell RNA-seq. (**D-E**) t-SNE plots illustrating YAP1 expression. (**F**) Validation of YAP1 mRNA expression in the *Allen Brain Atlas*

Based on the results obtained from bulk RNA-Seq, WGCNA analysis, and single-cell RNA-Seq, we identified 11 differentially expressed genes through Venn diagram analysis (Fig. 6A). PPI network analysis of these genes revealed YAP1 as the HUB gene, suggesting its pivotal role in mediating the development of depression (Fig. 6B). The remaining 10 genes may also play an important role in depression, and we will further investigate the changes in these genes in future studies. Furthermore, we examined the expression of YAP1 in normal individuals and patients with depression using the GSE32280 microarray dataset. We found that YAP1 expression was significantly elevated in patients with depression, corroborating our findings (Fig. 6C upper). In addition, we observed the expression level of YAP1 in single-cell sequencing data from human and macaque tissues, and found that YAP1 expression is relatively high in both species, suggesting that YAP1 may be a conserved gene (Fig. 6C lower). We investigated YAP1 expression in single-cell data from all cells and specifically in microglia. Our results indicated that YAP1 was predominantly expressed in cluster 1 of microglia (Fig. 6D-E). Subsequently, we observed YAP1 mRNA expression in the *Allen ISH brain atlas* and detected positive expression in the mouse prefrontal cortex, further validating our previous analysis (Fig. 6F).

### Correlation between YAP1 expression and HAMD scores in patients with depression

**Fig. 7:**
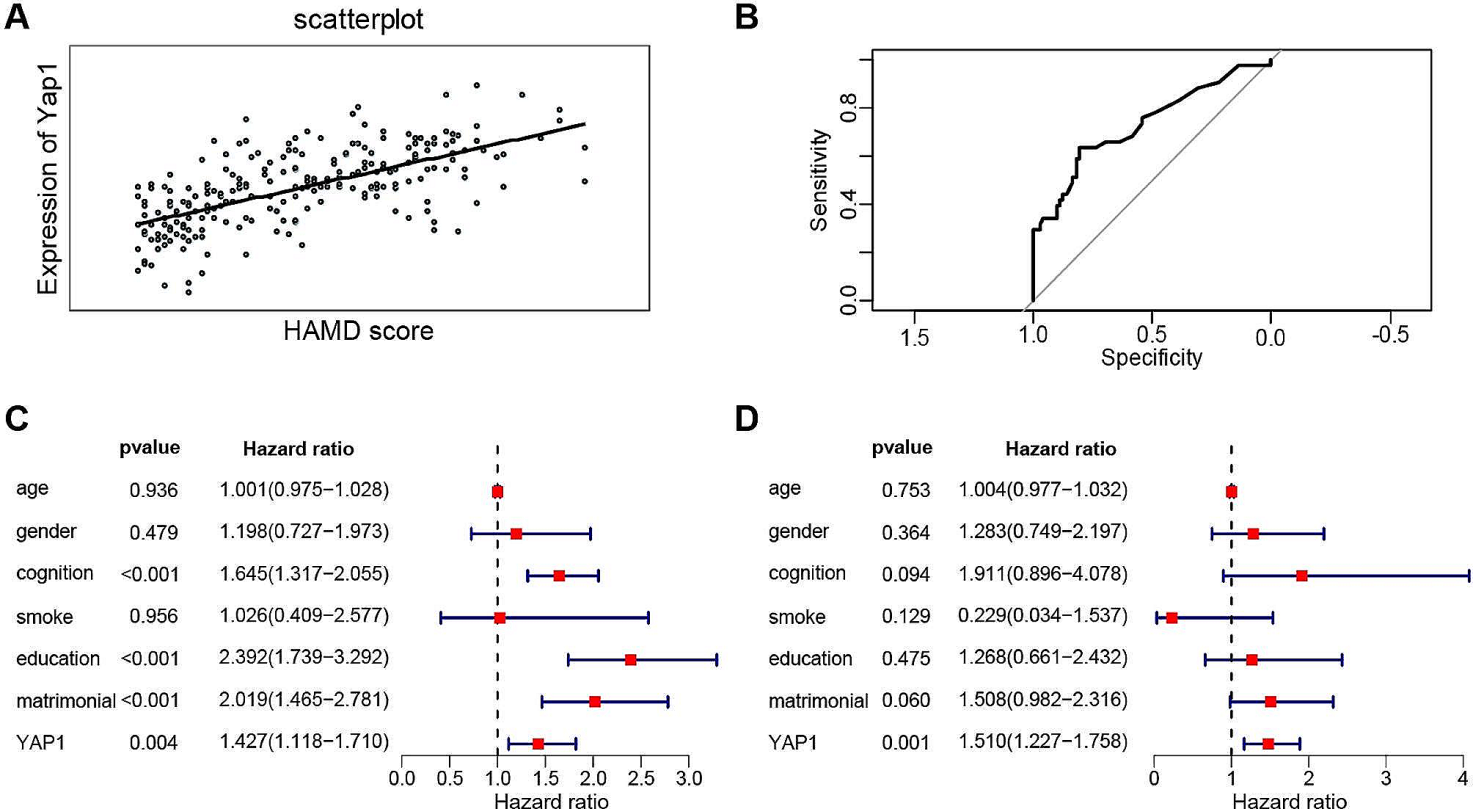
Correlation between YAP1 Expression and HAMD Scores in Depression Patients. (**A**) Scatter plot showing the relationship between YAP1 expression levels and HAMD scores. (**B**) ROC curve predicting depression based on YAP1 expression levels. (**C-D**) Univariate and multivariate analyses examining the relationship between YAP1 expression levels and depression

To further investigate the relationship between YAP1 expression levels and depression, we collected fasting venous blood samples from 159 patients with depression exhibiting varying degrees of severity. We measured serum YAP1 levels using ELISA and observed a positive correlation between HAMD scores (indicating depression severity) and YAP1 expression levels (Fig. 7A). ROC curve analysis suggested that YAP1 concentration could predict the occurrence of depression with good accuracy, with an AUC of 0.728, sensitivity of 78.2%, and specificity of 83.1% (Fig. 7B). Both univariate and multivariate analyses indicated that YAP1 expression level was an independent risk factor for depression (Fig. 7C-D) (*P* < 0.001).

## Discussion

In this study, we employed a combination of single-cell RNA-seq technology and bioinformatics analysis methods to delve into the role of microglia in the prefrontal cortex in the pathogenesis of depression, with a specific focus on the key molecule YAP1. Our findings suggest that microglia undergo significant changes in the prefrontal cortex of patients with depression, which may be closely associated with the onset of the disorder.

Through the analysis of single-cell RNA-seq data, we successfully categorized cells in the prefrontal cortex into distinct types and observed that microglia exhibited the most significant changes in proportion between normal and depressed states. This discovery aligns with previous research, emphasizing the importance of microglia in depression. As immune cells in the central nervous system, microglia play a crucial role in maintaining brain homeostasis and responding to various stimuli [18, 19]. Abnormal activation or dysfunction of these cells may lead to neuroinflammation and synaptic plasticity alterations, which are implicated in the pathogenesis of depression [20, 21]. Further sub-clustering analysis of microglia identified multiple distinct subpopulations, revealing significant differences between normal and depressed states. Differential gene expression analysis uncovered changes in several signaling pathways related to depression, including Rap1 signaling pathways, CAMP signaling pathways, and TGF-beta signaling pathways. These pathways are involved in neuroinflammation, synaptic plasticity, and neuronal survival, providing further support for the critical role of microglia in depression pathogenesis [22, 23].

Through interaction analysis and pseudotime analysis, we explored the interplay between different cell types in the prefrontal cortex and their gene expression changes across developmental states. These analyses uncovered alterations in ligand-receptor interactions and the activation or inhibition of various signaling pathways, offering new insights into depression mechanisms [24, 25]. Notably, STAT1 exhibited high differential expression levels across developmental states, likely due to its important role in neuroinflammation and immune responses [26, 27]. Cross-species analysis revealed significant correlations between microglia and inhibitory neurons in humans and rhesus macaques, emphasizing the importance of these cell types in depression. Additionally, we identified differences in microglia between the two species, such as CD family expression levels, which may provide new clues and potential therapeutic targets for human depression research [28, 29].

Moreover, through Bulk RNA-Seq and WGCNA analyses, we identified key genes associated with depression pathogenesis and discovered YAP1 as a central molecule. YAP1, a crucial transcriptional co-activator in the Hippo signaling pathway, plays a vital role in regulating cell proliferation, apoptosis, and organ size [30, 31]. Recently, increasing evidence suggests that YAP1 also has important functions in the nervous system, including regulating neuronal dendrite development, synaptic plasticity, and neural stem cell proliferation [10, 32]. Our findings indicate that YAP1 expression is significantly elevated in the prefrontal cortex of patients with depression, and its expression level positively correlates with depression severity. YAP1 may influence the development of neuroinflammation by regulating the activation state of microglia. For instance, a study on the optic nerve found that the activation of YAP1 can modulate the activation of microglia through the TGF-β signaling pathway, further affecting neuroinflammatory responses [33]. In Alzheimer’s disease (AD), YAP1 may interact with the HIPPO pathway, subsequently enhancing the ability of microglia to clear amyloid plaques, thus alleviating the symptoms of AD [34]. This suggests that YAP1 may serve as a potential therapeutic target for depression, offering new avenues for future drug development. Nevertheless, it should be noted that although this study found a possible correlation between serum YAP1 expression levels and HAMD scores, serum YAP1 levels may be influenced by various factors and may not truly reflect the expression situation in brain tissue. In future research, cerebrospinal fluid or prefrontal cortex tissue should be selected for related studies to improve the reliability of the research.

However, our study has some limitations. Firstly, although we utilized publicly available single-cell RNA-seq data for in-depth analysis, these data may be influenced by factors such as sample source, processing methods, and sequencing platforms, requiring cautious interpretation of the results. Secondly, our focus primarily centered on microglia in the prefrontal cortex, while the roles of other brain regions or cell types in depression pathogenesis remain to be further explored. Additionally, despite validating the expression of YAP1 through a multi-omics approach, a limitation of this study is that the effects of YAP1 knockdown or overexpression on microglia were not investigated at the cellular or animal level. We aim to address this issue in future research.

In conclusion, this study employed a combination of bioinformatics analysis methods and techniques to investigate the role of microglia in the prefrontal cortex in depression pathogenesis, with a particular emphasis on the key molecule YAP1. Our findings provide new perspectives and potential therapeutic targets for understanding depression pathogenesis, laying the foundation for future research.

### Electronic supplementary material

Below is the link to the electronic supplementary material.

Supplementary Material 1

## Data Availability

The raw data for this bioinformatics analysis all come from the GEO database, with specific accession numbers of GSE144136, GSE98793, GSE76826, GSE32280, and GSE201687. The code and data generated in this study are available from the corresponding author on reasonable request.
