# Supplementary figures and images for: Single-cell RNA-seq reveals the role of YAP1 in prefrontal cortex microglia in depression
Source: BMC Neurol. 2024 Jun 7;24:191. doi: 10.1186/s12883-024-03685-1 (PMC11157917; doi:10.1186/s12883-024-03685-1)

Pathway

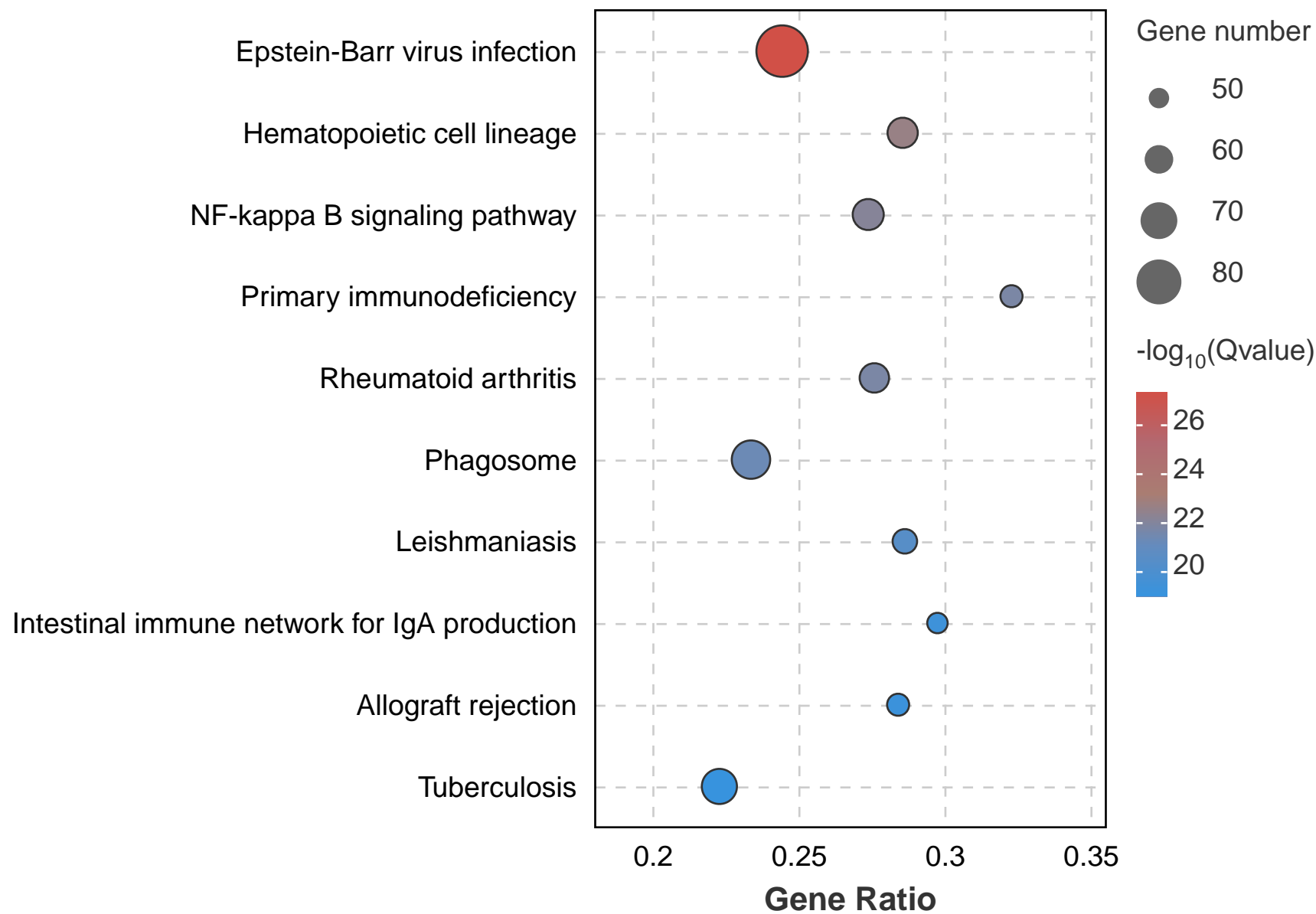

Supplement: Supplementary file 1 — Supplementary Material 1 [file 12883_2024_3685_MOESM1_ESM.pdf]
